# Chemical Fingerprinting of Biomass Burning Organic Aerosols from Sugar Cane Combustion: Complementary Findings from Field and Laboratory Studies

**DOI:** 10.1021/acsearthspacechem.3c00301

**Published:** 2024-02-28

**Authors:** Elena Hartner, Nadine Gawlitta, Thomas Gröger, Jürgen Orasche, Hendryk Czech, Genna-Leigh Geldenhuys, Gert Jakobi, Petri Tiitta, Pasi Yli-Pirilä, Miika Kortelainen, Olli Sippula, Patricia Forbes, Ralf Zimmermann

**Affiliations:** † Joint Mass Spectrometry Center (JMSC) at Comprehensive Molecular Analytics (CMA), Helmholtz Zentrum München, Ingolstädter Landstr. 1, D-85764 Neuherberg, Germany; ‡ Joint Mass Spectrometry Center (JMSC) at Analytical Chemistry, Institute of Chemistry, University of Rostock, Albert-Einstein-Straße 27, D-18059 Rostock, Germany; § Department of Chemistry, Faculty of Natural and Agricultural Sciences, University of Pretoria, Pretoria 0002, South Africa; ∥ Atmospheric Research Centre of Eastern Finland, Finnish Meteorological Institute, P.O. Box 1627, 70211 Kuopio, Finland; ⊥ Department of Environmental and Biological Sciences, University of Eastern Finland, Yliopistonranta 1, P.O. Box 1627, FI-70210 Kuopio, Finland; # Department of Chemistry, University of Eastern Finland, P.O. Box 111, FI-80101 Joensuu, Finland

**Keywords:** biomass burning, open-field burning, sugar cane, atmospheric aerosol, GC × GC-TOFMS, nontargeted analysis, combustion products

## Abstract

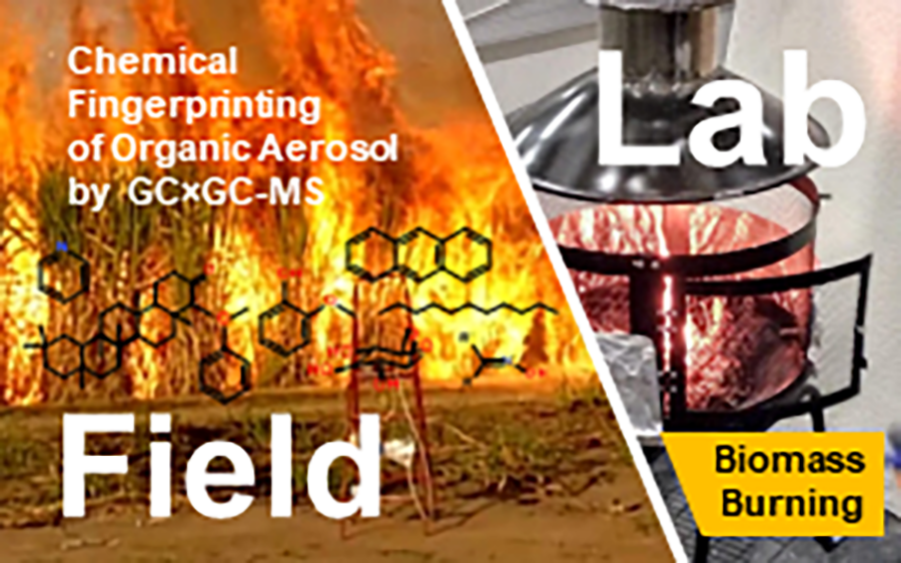

Agricultural fires are a major source of biomass-burning organic aerosols (BBOAs) with impacts on health, the environment, and climate. In this study, globally relevant BBOA emissions from the combustion of sugar cane in both field and laboratory experiments were analyzed using comprehensive two-dimensional gas chromatography time-of-flight mass spectrometry. The derived chemical fingerprints of fresh emissions were evaluated using targeted and nontargeted evaluation approaches. The open-field sugar cane burning experiments revealed the high chemical complexity of combustion emissions, including compounds derived from the pyrolysis of (hemi)cellulose, lignin, and further biomass, such as pyridine and oxime derivatives, methoxyphenols, and methoxybenzenes, as well as triterpenoids. In comparison, laboratory experiments could only partially model the complexity of real combustion events. Our results showed high variability between the conducted field and laboratory experiments, which we, among others, discuss in terms of differences in combustion conditions, fuel composition, and atmospheric processing. We conclude that both field and laboratory studies have their merits and should be applied complementarily. While field studies under real-world conditions are essential to assess the general impact on air quality, climate, and environment, laboratory studies are better suited to investigate specific emissions of different biomass types under controlled conditions.

## 1 Introduction

Agricultural fires are a common method for burning crop straw and crop residues around the world. These fires serve general purposes such as waste disposal (e.g., in order to clear farming land for agricultural use), as a control procedure for agricultural pests and diseases or as a management practice (e.g., in order to eliminate excess trash prior to harvesting).^1^

The open burning of biomass releases large amounts of air pollutants, with the largest proportion of emissions from open fires (80%) occurring in tropical and subtropical regions, particularly in (sub)tropical Africa and South America, and being largely anthropogenic in origin.^2^ Agricultural fires are also a significant source of air pollutants in China^3,4^ and Southeast Asia.^5^

Sugar cane (*Saccharum* spp.) is a crop widely cultivated throughout the (sub)tropical latitudes and serves as a food, animal feed, and energy crop.^6^ Before harvest, excess waste material such as leaves from the sugar cane plant has to be eliminated in order to facilitate the manual cutting of the stems with machetes and improve the efficiencies of harvesting and milling.^7^ This is commonly achieved by the open burning of sugar cane fields before harvesting. Due to the rising awareness of the adverse impacts of preharvest open-field fires, new laws and regulations have come into effect in some countries, such as Brazil, prohibiting the burning of sugar cane in areas where gentle slopes make green harvesting possible.^8^ Nonetheless, in South Africa, the preharvest burning of sugar cane is still widespread, and 90% of the cultivated sugar cane is burned.^9^

The large airborne quantities of pollutants released during the open combustion of sugar cane may cause haze events and widespread emission plumes with adverse health effects for residents and workers.^10,11^ Various atmospheric gases, such as carbon dioxide (CO_2_), methane (CH_4_), nitrous oxide (N_2_O), carbon monoxide (CO), nitrogen oxides (NO_*x*_), and volatile/semivolatile organic compounds (VOCs, SVOCs), are emitted.^12,13^ Furthermore, open biomass burning is the world’s largest atmospheric source of climatic effective black carbon (BC),^14^ accounting for 40% of global emissions.^2^ Additionally, organic aerosols (OAs) are the main component of particulate matter (PM) released from biomass combustion^15^ and, together with BC, are commonly referred to as carbonaceous aerosols. Biomass-burning OA (BBOA) emissions have adverse effects on the climate^13,16^ and the carbon cycle,^17,18^ as well as on human health.^19,20^ Thereby, both the physical properties (e.g., size, shape, and surface) and chemical properties (e.g., adsorbed compounds) of the PM have been shown to have a strong impact on its toxicity thereof.^21−24^ Among others, the physicochemical properties of the BBOA depend on various factors, such as the prevalence of smoldering or flaming combustion conditions, as well as environmental and meteorological conditions. The evaporation of PM, as well as the oxidation and condensation of emitted VOCs, additionally influences the chemical composition of the resulting aerosols,^25^ making biomass combustion a highly complex process.

To resolve the large number of constituents of highly complex OAs, thermal desorption comprehensive two-dimensional gas chromatography hyphenated with time-of-flight mass spectrometry (TD–GC × GC–TOFMS) has been shown to be useful for chemical fingerprinting of the (semi)volatile organic fraction in PM.^26^ As a powerful separation technique, GC × GC–MS has been used in several studies^15,27−29^ to effectively detect a wide range of compounds found in BBOAs. For the analysis of GC × GC–MS data, one typically differs between targeted and untargeted analysis. Whereas targeted analysis involves examining a preselected list of compounds, untargeted analysis entails evaluating all detectable compounds present in the sample.^30^ However, the untargeted assignment of significant marker compounds from biomass burning remains challenging due to the high complexity of environmental samples, which typically contain a large number of compounds. For these reasons, studies about BBOAs are often limited to specific target compounds, such as, for example, polycyclic aromatic hydrocarbons (PAHs), *n*-alkanes, or levoglucosan.

To deal with the high variability and chemical complexity of BBOAs, the simulation of atmospheric processes in simplified laboratory settings has become widely employed and has greatly contributed to our knowledge of the physiochemical properties of OA emissions. Other advantages of laboratory measurements include the use of more comprehensive instrumentation, the capacity to capture and sample all of the emissions from a complete burn, and the easier determination of fuel properties and elemental composition.^31^ Conversely, field studies better reflect real environmental conditions, actual fuel compositions, and fire behavior on a comparable scale, making them indicative of real fire scenarios.^31^ Integrating laboratory experiments with real-world field experiments poses several challenges due to differences in concentrations and temporal and spatial scaling, as well as the impact of different matrix effects on particle behavior.^25,32^

The scope of this study is an in-depth chemical characterization of organic carbonaceous aerosols in PM and the untargeted assignment of significant marker compounds arising from sugar cane burning emissions. For this purpose, offline samples from both laboratory combustion experiments and an open-field burning campaign in South Africa were comparatively investigated by TD–GC × GC–TOFMS. By combining observations from controlled biomass burning experiments in the laboratory with those from the field campaign, we aim to validate findings from laboratory studies and gain a more comprehensive understanding of biomass burning emissions under realistic environmental conditions.

## 2 Materials and Methods

### 2.1 Open-Burning Field Experiments

#### 2.1.1 Experimental Setup

In July/August 2018, open-field sugar cane fires were conducted on South African farms. The five sampling sites, located in two different regions in the province of Kwa-Zulu Natal in South Africa, have been described in detail by Geldenhuys et al.^7^ and are summarized in Table S1. Three burns were conducted on the north coast of Kwa-Zulu Natal, a coastal region with a warm and temperate climate. Two additional burns occurred around the Midlands of Kwa-Zulu Natal, which is located further inland and characterized by a more moderate maritime climate. Meteorological data were obtained from both stationary weather stations and a Kestrel 4500 pocket weather tracker (Kestrel Instruments, USA). Except for Burn_1, all fires were lit at the downwind edge of the field to ensure controlled burning of sugar cane at reduced speed.

#### 2.1.2 Sample Collection

During each experiment, a stationary sampling platform was installed downwind at approximately 16 m from the edge of the field. PM was collected on 47 mm quartz fiber (QF) filters (T293, Ahlstrom-Munksjö, Finland) for a sampling time between 10 and 20 min at a flow rate of 3.5 L min^–1^. Additionally, a background QF filter was sampled before fire initiation with a sampling volume of 35 L.

The gas phase was sampled by using adsorber tubes filled with graphitized carbon black (GCB). These gas-phase tubes consist of three layers of GCB sorbents designed to capture compounds with varying levels of volatility (Table S2). Sampling was done for 10 min at a flow rate of 0.5 L min^–1^, accounting for a total sampling volume of 5 L. All collected samples were stored at −20 °C until analysis.

Information about the sampling parameters for the collected QF filters and gas phase adsorber tubes is depicted in Tables S3 and S4, respectively.

### 2.2 Controlled Laboratory Experiments

#### 2.2.1 Experimental Setup

Laboratory experiments were performed in the aerosol physics, chemistry, and toxicology research unit (ILMARI) of the University of Eastern Finland. The sugar cane leaves used as fuel originated from both South African sampling sites (North Coast and Midlands of Kwa-Zulu Natal) to ensure maximum comparability. The dried whole leaves from both sites were burned in separate experiments. Open-fire combustion was simulated through the batchwise burning of 150 g of sugar cane leaves (5 × 30 g with 3 min per batch) in a specially designed combustion setup modified from an outdoor grill. In this setup, the biomass fuel is placed under a hood on a concave plate surrounded by metal mesh walls. The hood was connected to a chimney equipped with a flue gas fan, which drew the formed combustion exhaust emissions. Combustion air is directed through the mesh walls. The open space between the combustion setup and the hood provides additional air flow for immediate dilution and cooling of the formed exhaust aerosols, which is thought to resemble the typical conditions in the open-air burning of biomass. Each sugar cane sample was placed in a small steel mesh cage and ignited with a gas lighter. A partial sample of the exhaust aerosol was taken from the chimney, further diluted by a combination of porous tube-ejector diluters (by a factor of 6:1), and fed into the environmental chamber.^33^ In each experiment, samples from several combustion batches were collected into the chamber from where the samples for offline aerosol analysis were subsequently taken.

#### 2.2.2 Sample Collection

Both the particle and gas phases of BBOAs were sampled on QF filters and GCB adsorber tubes, respectively. As for the laboratory samples, the 47 mm QF filters (T293, Ahlstrom-Munksjö, Finland) were baked for 5 h at 550 °C, and the gas phase adsorber tubes were conditioned under a protective nitrogen atmosphere at 300 °C prior to sampling to remove possible organic contaminants. The PM was sampled for 30 min at a flow rate of 10 L min^–1^, accounting for a total sampling volume of 300 L. The gas phase was sampled for 30 min at a flow rate of 0.5 L min^–1^, accounting for a total sampling volume of 15 L. All samples were stored at −20 °C until analysis.

### 2.3 Instrumental Descriptions

#### 2.3.1 Particulate Matter

For the offline chemical characterization of the collected PM on QF filters, we applied TD–GC × GC–TOFMS on a Pegasus BT4D (LECO, St. Joseph, MI, USA) platform, equipped with an OPTIC-4 GC inlet system and a Cryofocus-4 cryogenic trapping system (GL Sciences, Eindhoven, Netherlands). For the analysis of the filter samples, a circular filter punch with diameters of 6 mm (field samples) and 10 mm (laboratory samples) was thermally desorbed in the injector and analyzed in a randomized order. For chromatographic separation, a nonpolar capillary column (60 m, BPX5, SGE, Australia) was installed in the first dimension and a midpolar capillary column (1.6 m, BPX50, SGE, Australia) in the second dimension (Table S5). Helium was used as a carrier gas. The mass spectrometric analysis occurred using electron ionization at 70 eV and detection by TOFMS. Details of the applied chromatographic and MS parameters for the analysis are provided in Tables S6 and S7, respectively. For further information on the temperature profiles during TD and cryogenic trapping, as well as on the flow parameters during analysis, refer to Figure S1.

For data acquisition and processing, ChromaTOF software version 5.5 (LECO, St. Joseph, MI, USA) was used. Furthermore, data evaluation and untargeted analysis were done with ChromaTOF Tile (v.1.2.6.0). The respective processing parameters are shown in Table S8.

#### 2.3.2 Gas Phase

TD–GC–MS was carried out with a TD-20 TD unit (Shimadzu, Japan), which was coupled to a GC–MS (GCMS-QP2010 Ultra, Shimadzu, Japan). The TD occurred at 345 °C for 45 min. Extracted compounds were first reconcentrated at 5 °C on a Tenax TD trap before being redesorbed at 330 °C for 30 min into the GC at a flow rate of 15 mL min-1 with a split ratio of 10 (field samples) and 2 (laboratory samples). The separation took place on a VF-xMS, high-arylene-modified phase column (30 + 5 m precolumn, 0.25 mm ID × 0.25 μm d*f*, Agilent Varian, USA). Further information on the temperature program of the GC, as well as the MS parameters for detection, is shown in Tables S9 and S10, respectively. Quantification of analyzed compounds was based on the internal standard calibration of certain compounds using an isotopically labeled internal standard and a calibration standard mixture (Table S11).

#### 2.3.3 Safety Statement

No unexpected or unusually high safety hazards were encountered.

## 3 Results and Discussion

This section introduces the general differences between the conducted field and laboratory experiments, such as differences in fuel composition, combustion conditions, and chemical processing after emission. Thereby, several combustion parameters are expected to influence the biomass burning emission characteristics such as the quality of combustion, the calorific value and moisture of the fuel, and the prevailing combustion phase.^34−38^ While solely dried sugar cane leaves were burned for the laboratory experiments, the field comprised a much more heterogeneous mix of materials, including the entire sugar cane plant (consisting of the stem, top, dry, and fresh leaves^39^) and other plants, as well as further organic material such as, e.g., insect pests, weeds, and biomass in the soil. In Table 1, a comparison of the properties of sugar cane leaves before and after the drying process is provided, which was done in preparation for the laboratory experiments. The data indicate an increase in the calorific value and a decrease in moisture following the drying procedure, which influence the combustion behavior.

**Table 1:** Properties and Elemental Characterization of Sugar Cane Leaves, Which Were Used as Fuel during the Laboratory Experiments; (qV, Gr) and (qp, Net) Refer to the Calorific Value at Constant Volume or Pressure; Values Were Determined by Eurofins Umwelt Ost GmbH (Bobritzsch-Hilbersdorf) Laboratory, Which Is Accredited According to DIN EN ISO/IEC 17025:2005 D-PL-14081-01-00

	Kwa-Zulu Natal Midlands		Kwa-Zulu Natal North Coast		
	original substance	dried substance	original substance	dried substance	unit
moisture	11.1		10.5		% (w/w)
gross calorific value (qV, gr)	17,300	19,500	17,200	19,300	kJ/kg
net calorific value (qp, net)	15,800	18,100	15,800	17,900	kJ/kg
ash content (550 °C)	2.3	2.6	2.4	2.6	% (w/w)
chlorine	0.044	0.05	0.115	0.129	% (w/w)
carbon	43.5	48.9	43.4	48.6	% (w/w)
hydrogen	5.5	6.2	5.5	6.2	% (w/w)
nitrogen	0.43	0.48	0.38	0.42	% (w/w)
sulfur	0.043	0.049	0.056	0.063	% (w/w)
oxygen	37.2	41.8	37.7	42.1	% (w/w)

Additionally, it is important to consider the distinction between the two main biomass combustion conditions: smoldering and flaming. Both can occur simultaneously in a fire and can alternate during burning, depending in a complex way on the availability and mixing of air with pyrolysis gases, temperature conditions, and fuel properties. Flaming is characterized by a high-temperature pyrolysis process with a relatively high combustion efficiency. As a fire progresses, smoldering combustion, which exhibits a lower combustion efficiency and produces high emissions of BBOAs, becomes increasingly prominent and involves surface oxidation and pyrolysis of both above- and below-ground biomass.^40^ It can be reasonably assumed that the open-field burning experiments, characterized by heterogeneous conditions, would likely lead to temporal and spatial variations in flaming and smoldering. Additional variables, which may influence biomass burning emissions, include the species composition, moisture content, and density of the biomass fuel, as well as meteorological parameters like wind.^25^ To best replicate the various stages of smoldering and flaming combustion observed during the open-burning of a sugar cane field, the laboratory experiments were conducted using a batchwise feeding approach for the sugar cane leaves as fuel. A study by Mugica-Álvarez et al.^41^ has shown that for better comparability of laboratory and field experiments, feeding in batches is preferable over continuous feeding since the latter leads to constant flaming conditions within the combustion chamber. Photographs of the conducted combustion experiments in both laboratory and open-field settings are shown in Figure S2.

Furthermore, the atmospheric lifetime of the resulting VOC and PM emissions needs to be considered. For instance, emissions of VOCs such as furans and phenolic compounds were observed to rapidly contribute to the formation of secondary OAs (SOAs) due to their short atmospheric lifetimes of ∼15–60 min (by reaction with OH^•^).^25,42^ In that regard, distinguishing between primary and secondary emissions proves challenging for field experiments, particularly for experiments such as Burn_2 and Burn_3, where the solar radiation exceeded 200 W m^–2^ (see Table S1). In such cases, rapid photooxidation cannot be ruled out, which makes it possible that during the field experiments, initial reactions such as evaporation and atmospheric aging may have already occurred before the first samples were collected approximately 10 min after fire initiation (Tables S3 and S4). Additionally, although dilution factors during the field experiments are unknown, the dilution of emission plumes may greatly impact gas–particle partitioning and evaporation.^25,43^ Lastly, differences in filter sampling conditions, such as variations in sampling time and flow rates, can also affect the partitioning of gas and particle phases through blow-on and blow-off effects. In that regard, in the field experiments, a sampling time of 10 min at 3.5 L min^–1^ was used, while in the laboratory experiments, the sampling time was extended to 30 min at 10 L min^–1^ to ensure sufficient filter loading for subsequent analysis. These differences in sampling conditions resulted in a lower filter loading for laboratory samples compared to that for the collected field samples.

### 3.1 Gas Phase

**Figure 1:**
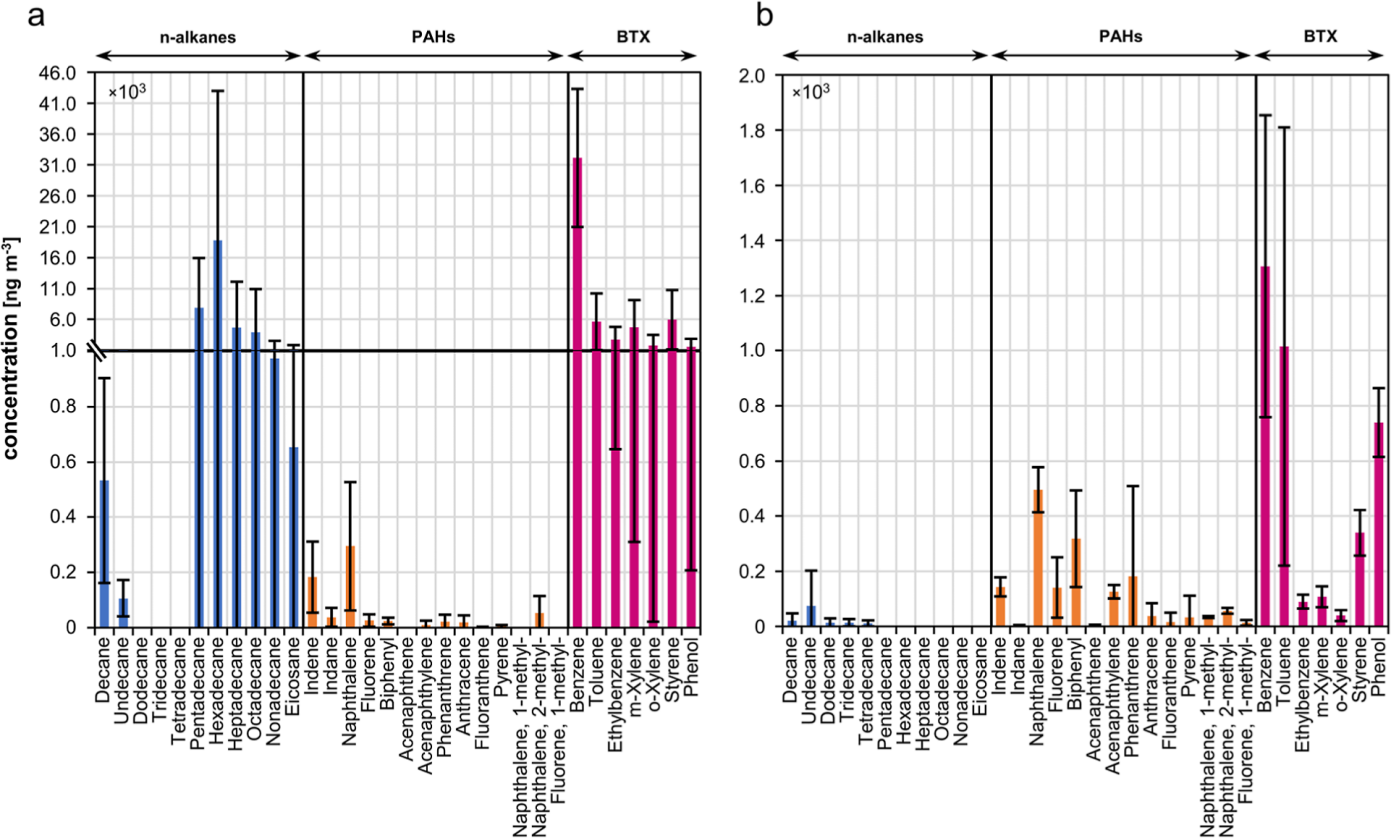
Quantification of gas phase components, namely, *n*-alkanes, (methylated) PAHs, and BTX from GCB gas phase tubes by TD–GC–MS for (A) field experiments and (B) laboratory experiments. Concentrations (ng m^–3^) were adjusted for blank and background levels and normalized to the corresponding sampling volume. Error bars represent the standard deviations (laboratory: *n* = 9; field: *n* = 5). The break in the *y*-axis at 1 × 10^3^ ng m^–3^ (only in plot A) indicates a nonlinear scale to better visualize low concentration levels in the bar chart.

The identification and quantification of VOCs and SVOCs in the gas phase were achieved via TD–GC–MS of the GCB gas phase tubes (Figure 1), with a focus on *n*-alkanes, PAHs, and a group of volatile aromatic hydrocarbons, namely, benzene, toluene, and xylene (BTX), consisting of (methylated or oxidized) benzene, toluene, and xylene compounds. Due to the lack of proper standardization, only the relative profiles of the emissions from the field and laboratory experiments can be compared.

The field samples exhibited high concentrations of *n*-alkanes and BTX aromatic compounds. In this respect, it is important to note that diesel emissions from agricultural machinery (e.g., tractor and water truck) were observed and that in one of the field experiments, a mixture of gasoline and diesel was used to start the fires (see Table S1), which may have contributed to the high emissions of alkanes and BTX. Furthermore, a mean concentration for total gas phase PAHs of 0.76 μg m^–3^ was found in the field samples. In comparison to our prior study by Geldenhuys et al.,^7^ where the mean total gas phase concentration of PAHs in the field experiments using a different sampling method with small polydimethylsiloxane portable denuder devices was reported at 3.4 μg m^–3^, the here presented analysis detected a lower total PAH content. Since this previous study revealed that 90% of the total PAHs were present in the gas phase rather than the particle phase, it is crucial to consider both the gas and particle phases to accurately account for gas–particle transformations occurring in the atmosphere. During the field experiments, a rapid dilution of the emission plume can be expected, leading to the evaporation of semivolatile compounds in the particle phase.

As a result, a comparison of absolute concentrations from the field and the laboratory is difficult. In addition, as described above, the field experiments are not fully traceable to the specific source and differ in the composition of the biomass fuel, e.g., in that the plant stalks and soil were also exposed to the fire. Nonetheless, when considering the benzene to toluene ratio (B/T), which is typically used to identify sources of VOC emissions but is also influenced by the underlying combustion conditions,^44^ there is a trend toward more flaming combustion in the field compared to the laboratory, as indicated by B/T ratios of 5.7 and 1.3 for the field and laboratory samples, respectively.

### 3.2 Particulate Phase

#### 3.2.1 Evaluation Based on Marker Compounds Found in Literature

We performed a targeted data evaluation of the filter measurements by TD–GC × GC–TOFMS for 64 compounds. These compounds have been previously identified in BBOAs according to the existing literature. However, we make no claim to completeness but rather aim to highlight the relative differences in chemical composition between the field and laboratory experiments. A list of the specific compounds, along with their respective literature sources, can be found in Table S12. The GC × GC contour plot in Figure S3a illustrates the positions of the peaks in the 2D chromatogram. Additionally, bubble plots for the mean abundances in laboratory and field experiments are depicted in Figure S3b,c, respectively.

**Figure 2:**
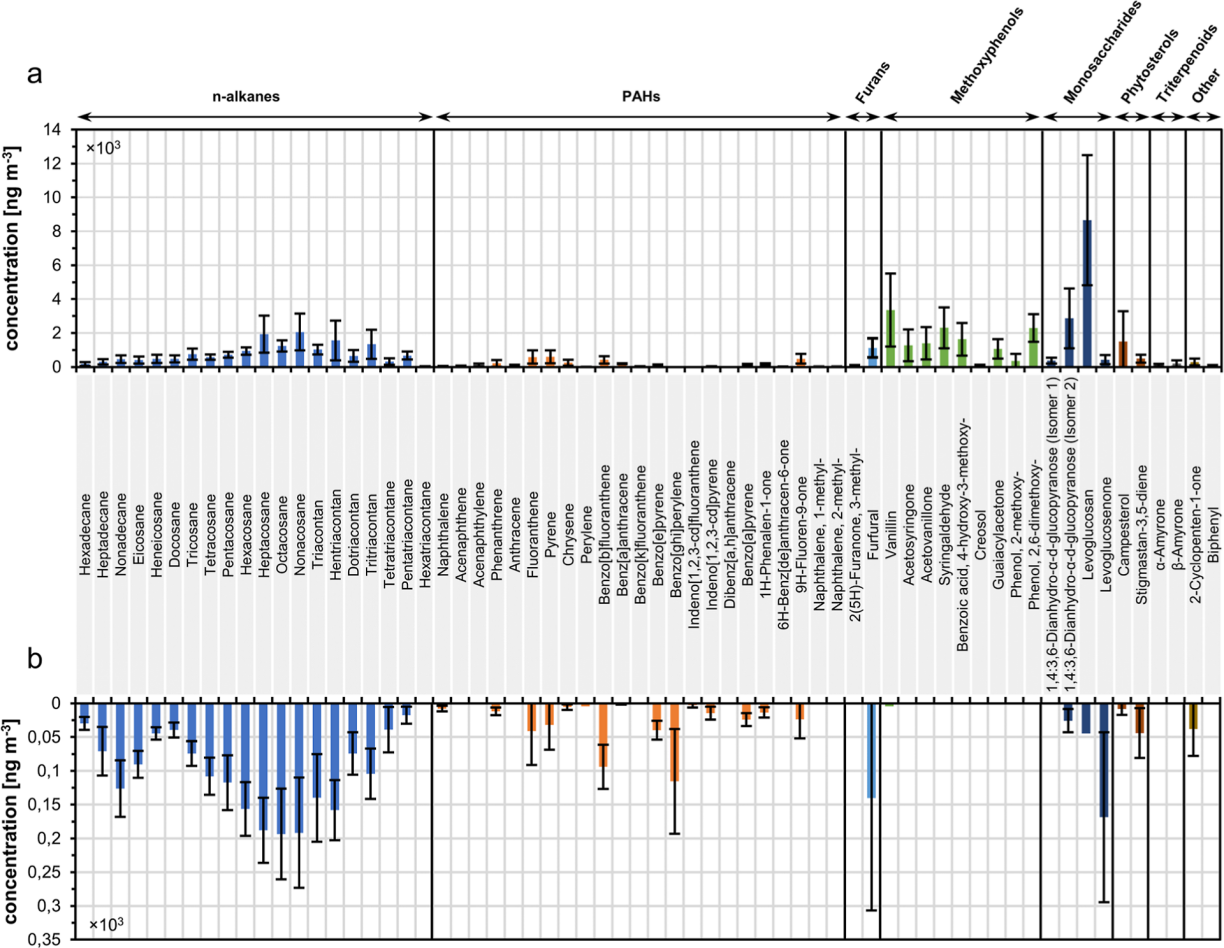
Semiquantification of the targeted reference particle phase compounds (see Table S12) from the (a) sugar cane burning field campaign in South Africa and the (b) laboratory combustion experiments. Each bar corresponds to the average concentration of a specific compound derived from TD–GC × GC–TOFMS measurements of the filter samples. Concentrations (ng m^–3^) were derived from a 4-point calibration using the internal standard compound fluorene-D10 and normalized to the corresponding sampling volume (see Figure S4). A tentative assignment of compounds to their respective molecular compositions was done with a NIST mass spectral library search (match quality ≥70%) and retention indices. Error bars represent the standard deviation values (laboratory: *n* = 9; field: *n* = 5).

Figure 2 illustrates the concentrations of these specific marker particle phase compounds associated with BBOAs found in both field (Figure 2a) and laboratory (Figure 2b) experiments. Semiquantification was performed using the internal standard compound fluorene-D10 (Figure S4). The target compounds have been categorized into eight different chemical classes, which include *n*-alkanes, PAHs [including oxygenated PAHs (O-PAHs) and methylated PAHs (M-PAHs)], furan derivatives, methoxyphenols, monosaccharide derivatives, phytosterols, triterpenoids, and other compounds.

In both types of experiments, a significant abundance of *n*-alkanes was found, which can be attributed to the naturally occurring leaf wax alkanes found in plants belonging to the grass family (Gramineae).^45^ We found a predominance for *n*-alkanes exhibiting an odd carbon number, which is typical for emissions derived from Gramineae.^46^ This is also reflected in the carbon preference index (CPI), which we calculated according to Simoneit^47^ as the ratio of *n*-alkanes (C_21_–C_34_) with odd and even carbon numbers. The resulting CPI values were greater than 1 for both field (1.7) and laboratory (1.2) samples. The highest concentration was observed for *n*-nonacosane (C_29_).

Among the PAHs detected in the laboratory experiments, the most dominant ones were benzo[ghi]perylene, benzo[*b*]fluoranthene, and fluoranthene. In the field experiments, the contribution of total PAHs to the total concentration of all 64 target compounds was less than that for the laboratory experiments, with the dominant PAHs being pyrene, fluoranthene, and benzo[*b*]fluoranthene.

Additionally, a significant presence of methoxyphenols was detected in the field samples. This included 2-methoxy-phenol, creosol, 2,6-dimethoxy-phenol, vanillin, vanillic acid, acetovanillone, guaiacylacetone, syringaldehyde, and acetosyringone. Except for vanillin, none of these compounds were detected in laboratory samples. Methoxyphenols are generated during biomass burning due to the pyrolysis of lignin, which has led to their widespread use as markers for BBOAs.^48^ They are considered important precursors for the formation of SOAs,^49^ with approximately 60% of the SOAs produced from biomass-burning emissions being attributed to the presence of oxygenated aromatic compounds including (methoxy-)phenols.^50^ Since these components are of great importance for the environmental impact of emissions from biomass combustion and a significant part of the chemical profile of open-field burning, it is notable that these compounds were not detected in the laboratory experiments. On the other hand, the sole presence of vanillin in small concentrations in the laboratory samples could show the initial state of emissions before further processing in the atmosphere. Lignin monomer ratios, such as the ratio of vanillic acid to vanillic aldehyde (vanillin) (VA_acid_/VA_al_), can be used as a measure of biomass burning plume aging. The field measurements give a value of 0.49 for VA_acid_/VA_al_, which is slightly above the typical range for fresh lignin-derived emissions of 0.1 to 0.2, which increases as atmospheric oxidation progresses.^51^ This process includes both the aqueous-phase photochemical oxidation and the direct photolysis of vanillin to form less volatile products.^52^ Thereby, the primary reaction product from the ozonolysis of vanillin is vanillic acid, which is formed through the functionalization of an aldehyde with a carboxylic acid group.^53^

We further observed notable emissions of furans, which are pyrolysis products of (hemi)cellulose and are emitted during both ignition and stable combustion phases.^54^ Thereby, variations in biomass composition may lead to different relative contributions of (hemi)cellulose-derived compounds, such as furans.^55^ For instance, Hatch et al.^56^ reported that the combustion of two different species of grasses (wiregrass and giant cutgrass) emitted different relative contributions of various groups of aromatic compounds. The detected elevated levels of furans in the emissions from wiregrass served as an indication for a higher cellulose content, while giant cutgrass exhibited a prevalence of benzenes, naphthalenes, and phenols, indicating a higher lignin content in the plant material. Indeed, although no differences were observed in the general composition of hemicellulose, cellulose, and lignin between roots, leaves, and stalks, clear differences were found in the specific hemicellulose sugar monomers and lignin monomers across different parts of the sugar cane plant.^57^ For instance, leaf tissues exhibited significantly higher levels of arabinose and galactose compared to those of stalk tissues,^57^ which could be an explanation for the observed differences in emissions from the laboratory and field experiments. Moreover, previous results by Geldenhuys et al.^7^ suggest that, in addition to differences in biomass composition and crop characteristics, the influence of the prevailing weather and combustion conditions on biomass combustion emissions and their gas–particle partitioning also play an important role in the emission profiles. In this respect, previous results showed clear differences in PAH emission profiles from one sugar cane burning event to another.^7^

The most prominent compound detected in the field samples was levoglucosan, which is derived from the thermal decomposition of cellulose and serves as a reliable marker for biomass burning. Interestingly, it was not detected in the laboratory samples, in which its dehydrated form levoglucosenone dominates instead. Other studies have also reported the occurrence of levoglucosenone in atmospheric OAs, even when levoglucosan was not detected.^58,59^ During the pyrolysis of cellulose, levoglucosenone is produced from levoglucosan through secondary pyrolysis reactions, including dehydration and fragmentation,^60^ which suggests that levoglucosenone may be directly emitted under certain burning conditions in the absence of water. This is consistent with the absence of levoglucosan in the laboratory experiments, where dried sugar cane leaves were burned. In contrast, during the field experiments, the burned leaves contained ∼10% moisture content (see Table 1) with additional water being present in the rest of the sugar cane plants and other organic material involved in the burning event. Furthermore, during Burn_4, which took place in the morning hours, morning dew could be observed, accounting for additional water presence.

#### 3.2.2 General Emission Profile Based on the 100 Most Abundant Compounds

**Figure 3:**
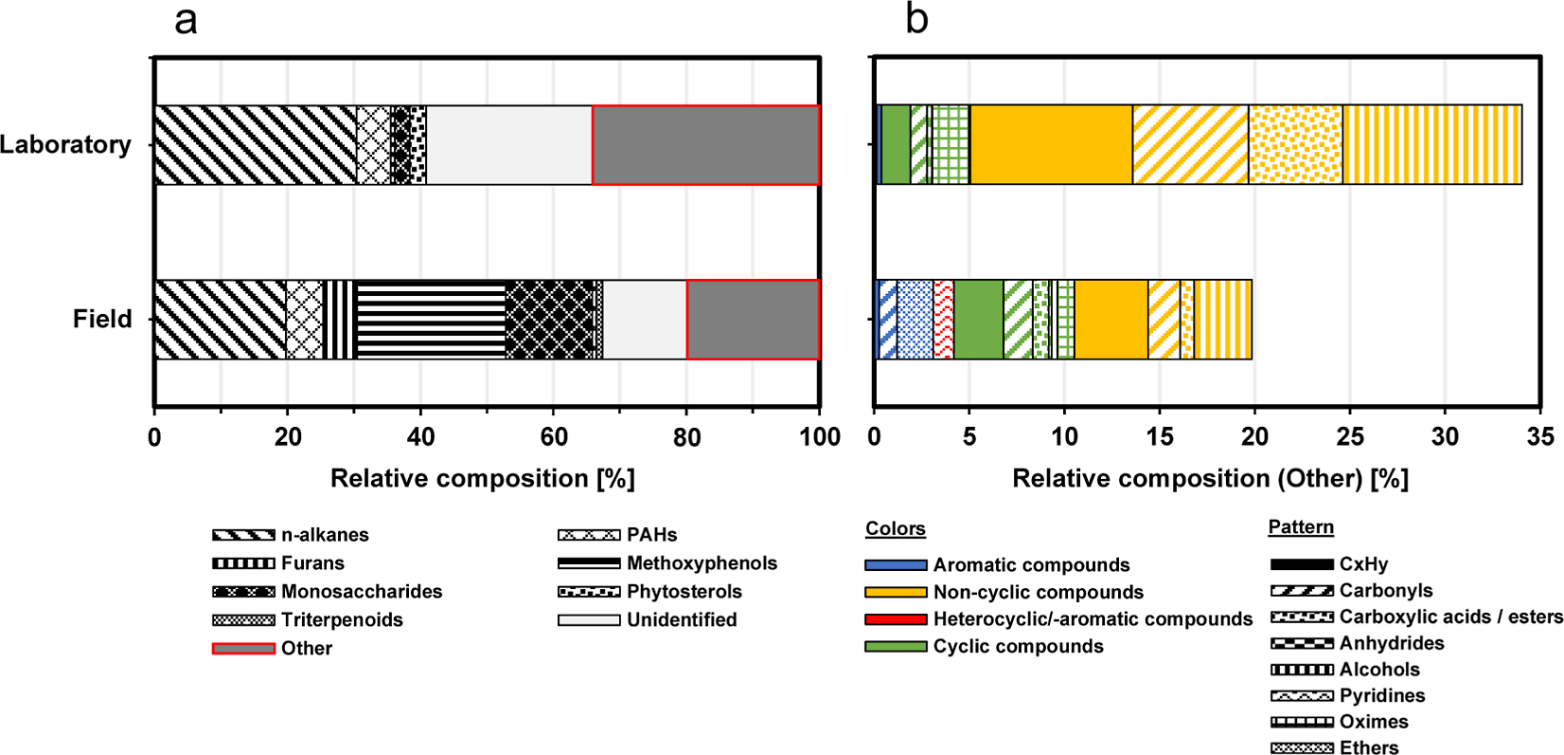
Bar charts illustrating the relative abundance of different compound classes for the 100 compounds with the highest signal intensities from TD–GC × GC–TOFMS measurements. The classified most abundant peaks contribute to 53.8 and 55.7% of the total TIC signal in the laboratory and field measurements, respectively. The tentative assignment of compounds to their respective molecular compositions was based on a NIST mass spectral library search (match quality ≥70%) and retention indices. (a) Categorization into eight chemical compound classes (*n*-alkanes, PAHs, furan derivatives, methoxyphenols, monosaccharide derivatives, phytosterols, triterpenoids, and unidentified compounds) based on the likewise classification of the previously discussed targeted reference compounds (see Figure 2). The bordered red bar illustrates the remaining compounds that could not be classified into these groups (other). (b) Subclassification of these “other” compounds into aromatic (blue), noncyclic (yellow), heterocyclic and heteroaromatic (red), and cyclic (green) compounds. The respective pattern illustrates a subclassification of the chemical functionalities.

Next, we shifted our attention to obtaining a broader perspective of sugar cane burning PM emissions by conducting a general overview of the 100 compounds with the highest signal intensities from both types of experiments. We classified these compounds into the same chemical classes as those in the previous section. The resulting relative composition (in terms of total ion chromatogram (TIC) peak area contributions) of these compound classes is shown in Figure 3a.

A notable distinction between the laboratory and field experiments is observed in the proportions of monosaccharide derivatives, furans, and methoxyphenols, which are higher in the field samples and align with observations in the preceding section. The fraction of compounds, which either did not belong to these classes or were unidentified, was 34.0 and 19.8% in the laboratory and field experiments, respectively. These compounds were further categorized into subclasses of compound classes and functional groups (Figure 3b).

While the relative contribution of PAHs was in a similar range for both laboratory and field samples, the latter exhibited a considerably higher abundance of aromatic species (3.1%) compared with that of the laboratory samples (0.4%). Also, the relative abundance of heterocyclic and heteroaromatic compounds was higher in the field samples (1.1%) compared to that of the laboratory samples (0.01‰). Thereby, for the field experiments, pyridines accounted for the entire contribution to the heterocyclic/-aromatic class. The significance of pyridines as markers for BBOAs will be discussed separately (Section 3.2.3.1). For both types of experiments, the relative contributions of cyclic compounds were comparable. However, in the laboratory samples, noncyclic compounds were found to be the predominant subclass, accounting for 29.0% of the relative composition. As previously described, these differences between field and laboratory experiments could again be attributed to variations in combustion conditions.

In addition to the higher prevalence of pyridines in the field samples mentioned above, there were notable differences in the abundance of certain functional groups and related compound classes, such as ethers, carboxylic acids, and oximes. The higher proportion of ethers in the field samples could entirely be attributed to a specific group of aromatic compounds known as methoxybenzenes. These compounds, like methoxyphenols, are compounds derived from the pyrolysis of lignin with considerable SOA formation potential.^61^ Conversely, the higher proportion of carboxylic acids in the laboratory samples could entirely be attributed to the class of noncyclic compounds. Interestingly, the presence of oximes in both laboratory and field samples can be solely ascribed to a single compound (4-(2,6,6-trimethyl-cyclohex-1-enyl)-but-3-en-2-one oxime). Oximes in plants, which are primarily derived from amino acids, are a highly diverse group of compounds in both their structure and function. Oximes have been shown to be emitted as VOCs and play a crucial role in plant communication, especially in response to herbivore or pest attacks.^62^ Moreover, they serve essential functions in plant growth and development. Despite their significance in plant metabolism, their exact functions and structures remain largely unknown and understudied. In fact, Sørensen et al.^62^ have suggested that various nontargeted metabolite profiling studies might have inadvertently detected oximes but failed to recognize them as such, dismissing them as experimental artifacts. It can therefore be assumed that there are many oximes that have yet to be discovered from various species of plants. Further studies are needed to verify whether the specific oxime derivative we discovered in this study can be specifically assigned to sugar cane. Given its detection among the most abundant 100 compounds in both field and laboratory settings, this makes it an interesting hypothesis for future investigations.

#### 3.2.3 Emission Profile Based on Individual Compounds Discovered by Nontargeted Analysis

Most target compounds, including levoglucosan and methoxyphenols, discussed earlier in this study, originate from the pyrolysis of biopolymers, such as cellulose and lignin. Hence, while these compounds are common markers for biomass burning, they are not specific to different biomass sources. Additionally, homologous series such as *n*-alkanes and (un)saturated carbohydrates substituted with functional groups like alcohols, carbonyls, carboxylic acids, or esters, as well as PAHs, are widely present in emissions from biomass burning. So far, these compounds cannot be attributed to a specific biomass source.^46^ Hence, nontargeted analysis was applied to identifying individual marker compounds.

**Figure 4:**
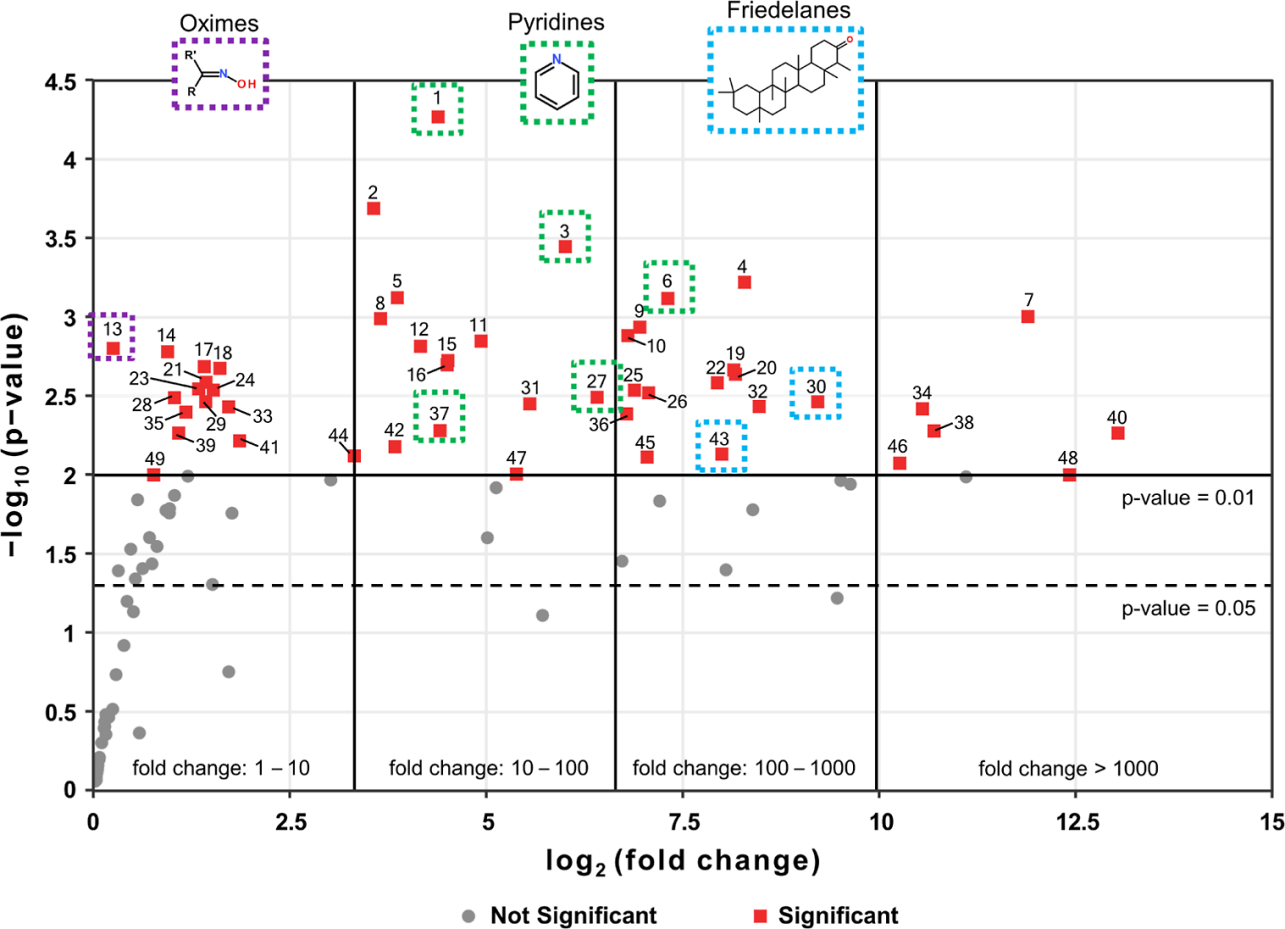
Volcano plot depicting the differential abundance of chemical compounds found by TD–GC × GC–TOFMS measurements of background and stationary filters from the field campaign, excluding unknowns, standard substances, and substances found in the background filter (sampled before fire initiation). The statistical significance (p-values from the Welch two-sample *t*-test) and fold changes were calculated using TIC areas, which were corrected for the respective sampling volume and normalized using fluorene-D10 as the internal standard (one-point normalization). The horizontal lines indicate the significance thresholds of p-values of 0.05 (black, dashed) and 0.01 (black). Compounds which surpass the p-value threshold of 0.01 (red rectangular markers) and exhibit increased abundance in comparison to the background filter measurements, as indicated by the vertical lines showing different fold change ranges, are discussed. The compound names for peaks 1–49 are provided in the Supporting Information (Table S13).

For the nontargeted analysis of the samples from the field experiments using TD–GC × GC–TOFMS, we applied a pixel-based evaluation approach using the ChromaTOF Tile software. Thereby, we only considered identified compounds with a NIST mass spectral library match quality ≥70% and compounds with mean areas greater than three times the standard deviation of the respective background. Subsequently, we employed a Welch two-sample *t*-test to assess the statistical significance of the abundance of compounds found in the plume of the burning sugar cane fields in comparison with the background measurements. The results are shown in a volcano plot (Figure 4). In total, we derived 49 compounds with positive fold changes and p-values below 0.01 (red rectangular markers), which are listed in Table S13 in increasing order based on their p-values.

Of the 49 compounds, a total of only 12 compounds, which include some PAHs, alkanes, methoxyphenols, and monosaccharides, have already been covered by the compounds found in the literature for targeted analysis (see Section 3.2.1). A Venn diagram representation of the overlap of chemical compounds derived from these two different evaluation approaches can be found in Figure S5. The compounds with the highest fold changes (>1000) were previously discussed methoxyphenols, such as syringic acid (40), acetosyringone (48), and 2,6-dimethoxyphenol (7). Compounds 38, 34, and 46 belong to the previously discussed compound classes of furan derivatives and (methoxy)benzenes.

We now discuss a selection of the individual compounds derived from Figure 4. While the complete list of all 49 compounds is available in Table S13, the compounds discussed in the main text are listed in Table 2. These compounds can be associated with the thermal breakdown of various bioactive substances and include pyridine and oxime derivatives (as briefly discussed in Section 3.2.2), as well as several friedelane triterpenoids.

**Table 2:**
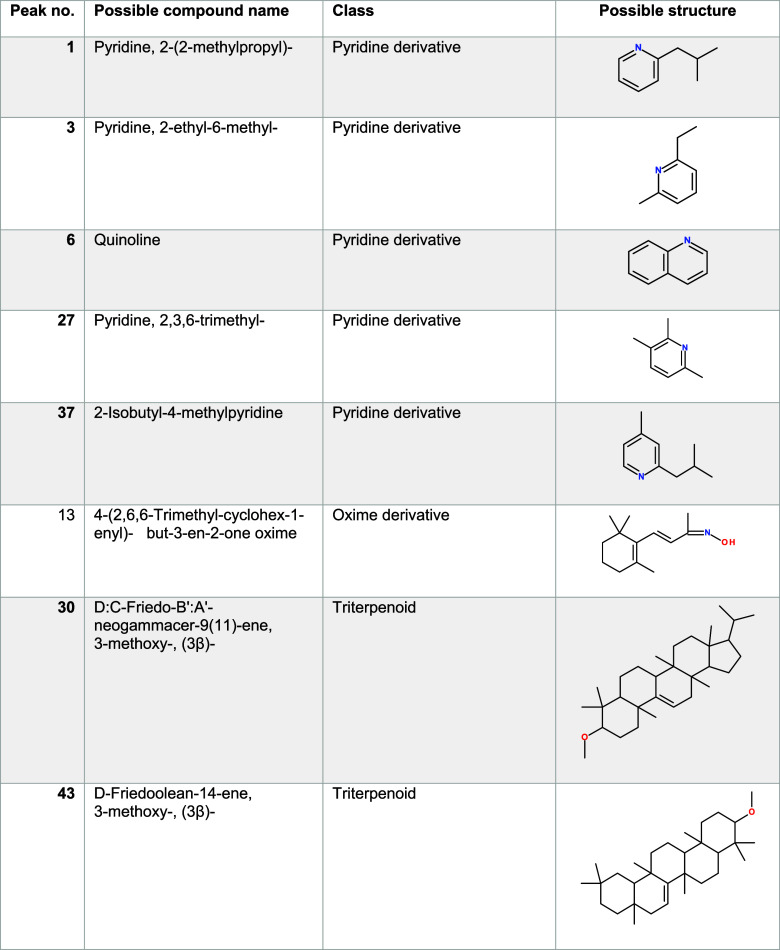
Selection of Compounds with Positive Fold Changes and *p*-values <0.01 Based on Figure 4, Which Are Discussed in the Text^a^

a The peak number refers to the numbers shown in the volcano plot (Figure 4). The complete list of the 49 compounds is provided in Table S13. Compounds were tentatively assigned to their respective molecular formulas via NIST mass spectral library search (match quality ≥70%) and retention indices.

##### 3.2.3.1 Pyridine Derivatives

Nitrogen-containing heteroaromatics such as (alkyl)pyridines and quinoline (peak numbers 1, 3, 6, 27, and 37) were present at high fold changes and significance in the emission plume from the field burning experiments, as evident from Figure 4 and Table 2. Generally, nitrogen-containing heterocycles are attributed to the pyrolysis of nitrogen-rich components found in vegetation, such as amino acids, proteins, and other biopolymers.^63^ As an example, the formation of (alkyl-)pyridines has been attributed to the pyrolysis of the amino acids α- and β-alanine.^64^ Furthermore, nitrogen-containing heterocycles occur naturally in biomass as they are not only synthesized by plants but also by bacteria, fungi, and other living organisms.^65^

According to Kosyakov et al.,^66^ who identified pyridine derivatives from peat burning emissions, they are predominantly formed under oxygen-deficient conditions and at temperatures around 500 °C. Nitrogen-heteroaromatics, including pyridine derivatives, have further been reported for rice straw burning emissions using GC × GC.^63^ Also, Hatch et al.^56^ have reported pyridine, 2-methyl-pyridine, and 3-methyl-pyridine from biomass burning emissions, whereas the highest relative contribution from nitrogen-containing species has been found in the smoke from grassland emissions (giant cutgrass) compared to those of other fuels such as spruce, pine, rice straw, and peat. Nitrogen-containing heteroaromatics have been found to exhibit high thermal stability comparable to their polyaromatic counterparts lacking heteroatoms.^67^

Our findings suggest that agricultural fires, such as open-field sugar cane burns, could be a significant and often overlooked source of (alkyl-)pyridines released into the atmosphere. There are not many scientific publications about pyridines in environmental samples since they are commonly not part of targeted analysis techniques despite posing a potential emission problem due to their high stability and ability to be transported over long distances. This underscores the importance of employing nontargeted evaluation methods, which analyze the entire chemical composition and allow for a comprehensive assessment of biomass burning emissions. Considering that the dried sugar cane leaves were found to contain approximately 0.45% (w/w) nitrogen content (see Table 1), it is crucial to include compounds in chemical analysis that may exist in small concentrations or have not been previously studied.

##### 3.2.3.2 Oxime Derivatives

The listed oxime derivative (peak number 13) has already been discussed (Section 3.2.2) as a compound present in high abundance in both field and laboratory samples. Also, for the nontargeted evaluation deducted here, 4-(2,6,6-trimethyl-cyclohex-1-enyl)-but-3-en-2-one oxime was revealed to be a relevant compound and exhibited a low p-value (0.0016), which suggests a significant difference between the background and plume measurements during the field experiments. However, the relatively low fold change (1.2) indicates that the implication of this finding might be relatively small in practical terms, and therefore, further validation is necessary to establish the significance of this finding.

##### 3.2.3.3 Friedelane Triterpenoids

While friedelane triterpenoids (peak numbers 30 and 43) have been extracted from cork and other plants^68−70^ and have also been found in the biomass burning smoke of certain conifers,^71^ they are not commonly observed in Gramineae. However, in a recent study by Radi et al.,^72^ friedelane triterpenoids and other pentacyclic triterpenoids were identified in the shavings of bamboo bark, which does fall under the Gramineae family. Additional research is necessary to explore whether the observed friedelane triterpenoids in the open-field burning experiments could originate from their presence in the stems of sugar cane. This could potentially explain the absence of such compounds in our laboratory experiments where only the leaves were burned.

## 4 Conclusions

In conclusion, this study presents a comprehensive comparison of laboratory and field experiments on biomass burning, specifically focusing on the gas-phase and particle-bound chemical emissions derived from the combustion of sugar cane leaves. The experiments highlighted the chemical complexity and variability of biomass burning conditions and primary emissions, which are influenced by numerous factors such as fuel composition, combustion conditions, and possible postemission atmospheric processing.

We found that the conducted laboratory experiments may not fully represent real-world scenarios and reflect only part of what we found in the field experiments. However, the controlled combustion of selected biomass allows for the controlled isolation of the target matrix. This might be especially useful for clarifying primary marker compounds that are not influenced by subsequent atmospheric processing or emissions from other artificial sources, which are not primarily associated with the burning of the pure matrix.

The laboratory experiments involved burning dried sugar cane leaves, while the field experiments encompassed a more diverse mix of biomass. This heterogeneity, combined with the dynamic nature of biomass burning, introduces a significant degree of variability in observed emissions. The study further underscored the importance of considering both flaming and smoldering combustion conditions, which alternate and co-occur during a fire, influencing the emission profiles.

While open-field burning experiments provide a more realistic setting, they are influenced by uncontrolled variables. However, since real-world burning events account for these unpredictable factors, field studies are essential for understanding the environmental, climatic, and health effects of biomass burning emissions. Consequently, both approaches are valuable and complementary.

In terms of emission composition, the study identified numerous VOCs and particulate-bound SVOCs, with significant differences observed in the emission profiles between field and laboratory experiments.

Chemical markers for biomass burning, including *n*-alkanes, PAHs, furan derivatives, and methoxyphenols, were detected in both types of experiments. However, notable differences were observed in the relative abundances of these compounds. For instance, methoxyphenols, which are produced during the pyrolysis of lignin and serve as important precursors for SOA formation, were significantly more abundant in the field samples, which could be explained due to different burning conditions but also possible short-term postemission processes like photo-oxidation. Dilution of emission plumes, variations in sampling conditions, and atmospheric aging can further influence the observed emission profiles. In this context, the study highlighted the need to consider both gas and particle phases of emissions to accurately account for the gas–particle ratio in the atmosphere vs closed laboratory experiments.

Besides general emission profiles for compounds, we were also able to identify unique compound groups that have not been associated or discussed with sugar cane burning so far.

This study offers valuable insights into the complex dynamics of biomass burning and its emissions. It underscores the need for careful consideration of combustion conditions, fuel composition, and postemission processes when studying and modeling biomass burning emissions. The findings also highlight the challenges in comparing laboratory and field experiments, given the inherent variability and complexity of real-world biomass burning events. This work contributes to a more nuanced understanding of biomass burning emissions, paving the way for more accurate emission inventories and improved atmospheric models.

## Supporting Information Available

Experimental conditions during the five open-field sugar cane burning experiments in South Africa; composition of the graphitized carbon black gas phase sampling tubes; sampling parameters for collected particulate matter and gas phase during the open-field sugar cane burning experiments; column setup for analysis of PM by TD–GC × GC–TOFMS; GC and MS parameters for analysis of PM by TD–GC × GC–TOFMS; temperature and flow settings for the OPTIC-4 inlet and cold trap system for analysis of PM by TD–GC × GC–TOFMS; data processing parameters for evaluation of TD–GC × GC–TOFMS data with ChromaTOF Tile; GC and MS parameters for gas phase analysis GC–MS; overview of compounds used for the internal and calibration standard mixtures for gas phase analysis by GC–MS; photographs of the combustion of sugar cane in the laboratory and in the field experiments; list of key marker compounds found biomass burning aerosols according to literature, which we used for targeted evaluation of PM; GC × GC contour and bubble plots of these targeted key marker compounds; information on performed semiquantification of particle phase compounds by TD–GC × GC–TOFMS using the internal standard compound fluorene-D10; list of compounds derived from nontargeted analysis of sugar cane burning emissions in the field experiments; and Venn diagram of compounds derived from targeted and nontargeted evaluation of sugar cane burning emissions PDF
