# Supplementary material for: Chemical Fingerprinting of Biomass Burning Organic Aerosols from Sugar Cane Combustion: Complementary Findings from Field and Laboratory Studies
Source: ACS Earth Space Chem. 2024 Feb 28;8(3):533–46. doi: 10.1021/acsearthspacechem.3c00301 (PMC10961841; doi:10.1021/acsearthspacechem.3c00301)
Supplement: Supplementary file 1 — sp3c00301_si_001.pdf [file sp3c00301_si_001.pdf]

Supplementary Information

Chemical Fingerprinting of Biomass Burning Organic Aerosols from Sugar Cane Combustion: Complementary Findings from Field and Laboratory Studies

Elena Hartner[1,2], Nadine Gawlitta[1], Thomas Gröger[1,], Jürgen Orasche[1], Hendryk Czech[1,2], Genna-Leigh Geldenhuys[3], Gert Jakobi[1], Petri Tiitta[4], Pasi Yli-Pirilä[5], Miika Kortelainen[5],Olli Sippula[5,6], Patricia Forbes[3], Ralf Zimmermann[1,2]*

*Corresponding author: thomas.groeger@helmholtz-munich.de

[1] Joint Mass Spectrometry Center (JMSC) at Comprehensive Molecular Analytics (CMA), Helmholtz Zentrum München, Ingolstädter Landstr. 1, D-85764 Neuherberg, Germany

[2] Joint Mass Spectrometry Center (JMSC) at Analytical Chemistry, Institute of Chemistry, University of Rostock, Albert-Einstein-Straße 27, D-18059 Rostock, Germany

[3] Department of Chemistry, Faculty of Natural and Agricultural Sciences, University of Pretoria, Pretoria 0002, South Africa

[4] Atmospheric Research Centre of Eastern Finland, Finnish Meteorological Institute, P.O. Box 1627, 70211 Kuopio, Finland.

[5] Department of Environmental and Biological Sciences, University of Eastern Finland, Yliopistonranta 1, P.O.Box 1627, FI-70210 Kuopio, Finland

[6] Department of Chemistry, University of Eastern Finland, P.O.Box 111, FI-80101 Joensuu, Finland

Content

Table S1: Experimental conditions during the five open-field sugarcane burning experiments in South Africa

Table S2: Composition of the graphitized carbon black (GCB) gas phase sampling tubes
Table S3: Sampling parameters for collected particulate matter (PM) during the open-field sugarcane burning experiments
Table S4: Sampling parameters for collected gas phase during the open-field sugarcane burning experiments
Table S5: Column setup for analysis of PM by TD–GC×GC–TOFMS
Table S6: GC temperature profile for analysis of PM by TD–GC×GC–TOFMS
Table S7: MS parameters for analysis of PM by TD–GC×GC–TOFMS
Figure S1: Temperature and flow settings for the OPTIC-4 inlet and cold trap system for analysis of PM by TD–GC×GC–TOFMS
Table S8: Data processing parameters for evaluation of TD–GC×GC–TOFMS data with ChromaTOF Tile
Table S9: GC temperature profile for gas phase analysis by GC–MS
Table S10: MS parameters for gas phase analysis by GC–MS
Table S11: Overview of compounds used for the internal and calibration standard mixture for gas phase analysis by GC–MS
Figure S2: Photographs of the combustion of sugarcane in the laboratory and in the field experiments
Table S12: List key tracer compounds found biomass burning aerosols according to literature, which we used for targeted evaluation of PM
Figure S3: GC×GC contour and bubble plots of these targeted key tracer compounds
Figure S4: Semi-quantification of particle phase compounds by TD-GC×GC-TOFMS using a 4-point calibration of the internal standard compound fluorene-D10
Table S13: List of compounds derived from non-targeted analysis of sugarcane burning emissions in the field experiments
Figure S5: Venn diagram of compounds derived from targeted and non-targeted evaluation of sugarcane burning emissions

***Table S 1:** Experimental conditions during the five open-field sugarcane burn events conducted, including details on the sampling location, sugarcane crops, meteorological parameters and general observations during the experiments (as published by Geldenhuys, et al. [1]).*

Experiment Name	Burn_1	Burn_2	Burn_3	Burn_4	Burn_5
Sampling Location	Kwa-Zulu Natal North Coast 29° 17′ 42.7″S	Kwa-Zulu Natal North Coast 29° 01′ 41.1″S	Kwa-Zulu Natal Midlands 29° 20′ 56.7″S	Kwa-Zulu Natal Midlands 29° 21′ 09.4″S	Kwa-Zulu Natal Midlands 29° 21′ 17.4″S

	31° 16' 13.1″E	31° 36′ 31.9″E	30° 46′ 05.8″E	30° 46′ 19.3″E	30° 46′ 16.5″E
**Altitude (m)**	170	60	942	888	882
**Crop type (age)**	N58<br>(14 months)	Mixed<br>(18 months)	N42<br>(20 months)	N57<br>(n.a.)	N58<br>(20 months)
**Date and time of burn event**	180730 (AM)<br>7:31	180730 (PM)<br>15:45	180801 (PM)<br>15:55	180802 (AM)<br>7:45	180802 (PM)<br>15:07
**Observations during burn**	Ignition upwind; Fast and hot burn	Ignition downwind; Slow burn	Ignition downwind (with gasoline/diesel); Fast burn due to very dry and dense crop; Lots of black particles in the plume	Ignition downwind; Fast burn; Plume went straight upwards and dispersed fast; Little smoke	Ignition downwind; Slow burn
**Weather observations**	Cloudy and cool, gusty winds	Clear sky, gusty winds	Cloudy, windy	Partly cloudy, no wind, still conditions, morning dew	Cloudy, cool, gusty winds
**Temperature (°C)**	22.3	22.5	23.6	10.0	10.1
**Humidity (%)**	66.5	59.2	54.1	94.3	84.5
**Solar Radiation ($W\ m^{-2}$)**	29.4	370.8	208	38	165.7
**Wind velocity ($m\ s^{-1}$) and wind direction**	1.6<br>W	4.4<br>SSW	2.4<br>SSE	0.1<br>W	3.5<br>S

Table S 2: *Composition of gas phase sampling tubes containing graphitized carbon black (GCB) (Merck, Germany) as sorbent material. Each gas phase sampling tube was composed of three different GCB layers separated by glass wool. Thermal desorption was done with reversed airflow compared to sampling.*

Sampling order in direction of airflow	Desorption order in direction of airflow	Sorbent	Weight [mg]
1	3	Carbotrap® B (20-40 mesh)	60
2	2	Carbotrap® Y (20-40 mesh)	60
3	1	Carboxen® 569 (20-45 mesh)	60

Table S 3: *Sampling parameters for the collected particulate matter on quartz fiber (QF) filters (T293, Ahlstrom-Munksjö, Finland). Prior to sampling, the QF filters were baked for 5 h at 550 °C to remove possible organic contaminants.*

QF filters	Sample	Start time	Flow [$L\ min^{-1}$]	Time [min]	Sampling volume [L]
**Burn_1**	Background	07:00	3.5	10	35
	Plume	07:31	3.5	10	35
**Burn_2**	Plume	15:44	3.5	10	35

**Burn_3**	Background	15:37	3.5	10	35
	Plume	15:56	3.5	19	66.5
**Burn_4**	Background	07:20	3.5	10	35
	Plume	07:45	3.5	10	35
**Burn_5**	Background	15:32	3.5	10	35
	Plume	15:22	3.5	16	56

Table S 4: *Sampling parameters for the collected gas phase on GCB adsorber tubes. Prior to sampling, the gas phase adsorber tubes were conditioned under a protective nitrogen atmosphere at 300 °C to remove possible organic contaminants.*

Gas phase tubes	Sample	Start time	Flow [L min^{-1}]	Time [min]	Sampling volume [L]
**Burn_1**	Background	07:00	0.5	10	5.0
	Plume	07:31	0.5	10	5.1
**Burn_2**	Background	15:08	0.5	10	5.0
	Plume	15:44	0.5	10	5.0
**Burn_3**	Background	15:37	0.5	10	5.0
	Plume	15:56	0.5	11	5.3
**Burn_4**	Background	07:20	0.5	10	5.0
	Plume	07:45	0.5	10	5.0
**Burn_5**	Background	15:32	0.5	10	5.0
	Plume	15:22	0.5	16	8.0

Table S 5: *Column setup for TD-GC×GC-TOFMS analysis of filter samples.*

Column setup	Type	Length [m]
Pre-column	SGE™ BPX5<br>(5 % phenyl polysilphenylene-siloxane,<br>0.25 mm i.d., 0.25 µm df, SGE, Australia)	1.3
1st Dimension	SGE™ BPX5<br>(5 % phenyl polysilphenylene-siloxane,<br>0.25 mm i.d., 0.25 µm df, SGE, Australia)	60
2nd Dimension	SGE™ BPX50	1.6

	(50 % phenyl polysilphenylene-siloxane, 0.1 mm i.d., 0.1 µm df, SGE, Australia)	

Table S 6: *Primary GC oven temperature profile for TD-GC×GC-TOFMS analysis of filter samples. The secondary oven was offset by +20 °C relative to the primary oven temperature and the modulator was offset by +15 °C relative to the secondary oven temperature. The modulation time was 3 sec with a hot pulse time of 1.5 sec.*

T rate [°C min^{-1}]	T [°C]	Holding time [min]
-	40	10
2	250	-
5	330	10

Table S 7: *Mass spectrometric parameters for TD-GC×GC-TOFMS analysis of filter samples.*

MS Parameter	Setting
Transfer line temperature	300 °C
Acquisition rate	100 spectra sec^{-1}
Mass acquisition range	20 – 700 Daltons (Da)
Electron ionization energy	70 eV
Ion source temperature	250 °C

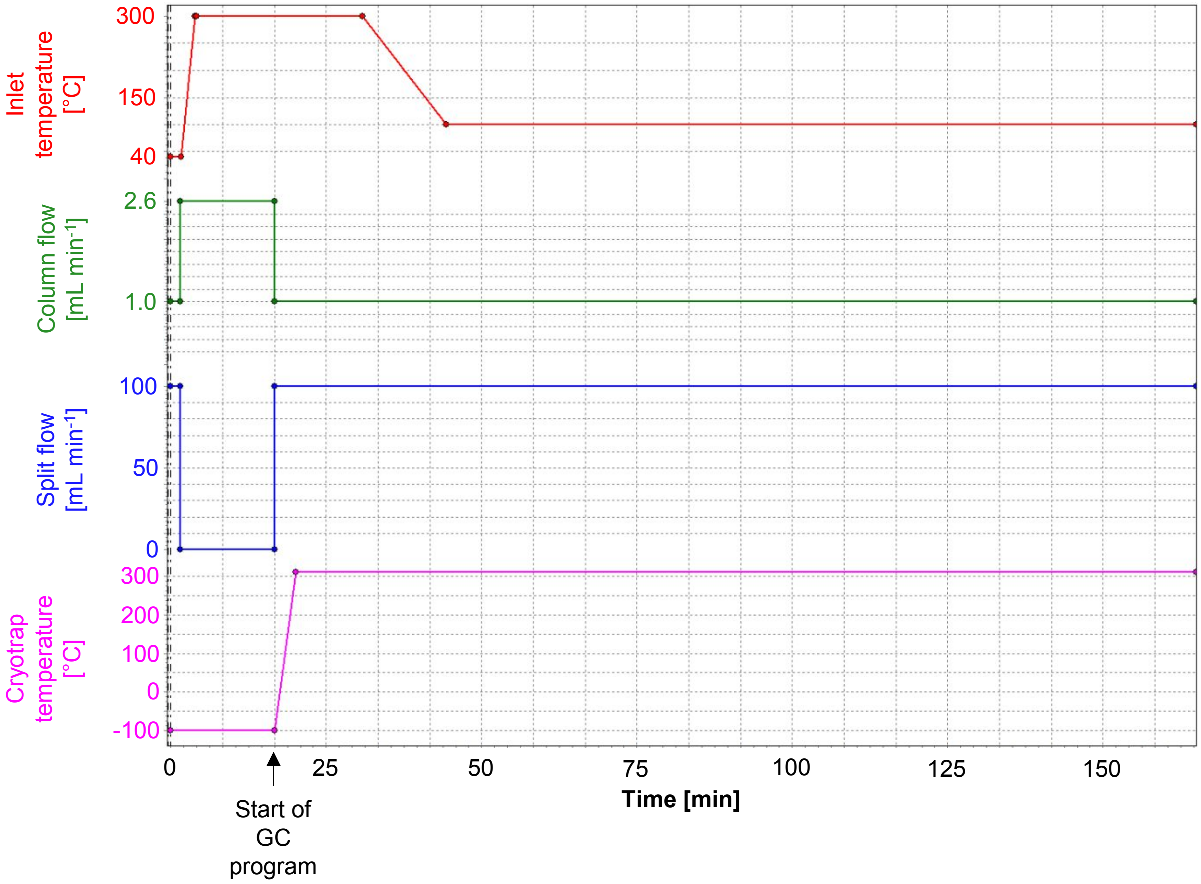

Figure S 1: *Temperature profile (red), column flow (green) and split flow (blue) parameters for OPTIC-4 inlet system (GL Sciences, Netherlands), as well as temperature profile of Cryofocus-4 cold trap system (pink) (GL Sciences, Netherlands) for TD-GC×GC-TOFMS analysis. The thermal desorption of the SVOC from a QF filter punch (d=10 mm) occurred through the application of a gradual thermal gradient of 2 °C sec^{-1} from 40 °C to 300 °C in order to introduce the analytes onto the GC column set. The inlet purge time was 100 sec at a column flow of 1 mL min^{-1} and a split flow of 100 mL min^{-1}. The desorption flow rate was 2.6 mL min^{-1} in spitless mode. Subsequently, the thermally desorbed analytes were cryogenically focused with the cryotrap at −100 °C. Following the thermal desorption, the column flow was reduced to 1 mL min^{-1} and the split flow increased to 100 mL min^{-1}.*

Table S 8: *Data processing parameters for ChromaTOF Tile (Version v.1.2.6.0, LECO, USA)*

Parameter	Detail
Tile size D1 (modulations)	4
Tile size D2 (spectra)	31
S/N threshold	1000
Samples that must exceed S/N threshold	3
Mass F-ratios to average	3
Threshold type to apply	F-ratio
F-ratio threshold	0
Minimum masses per tile	3
Minimum mass	0
Maximum mass	600
Masses to ignore	73; 147
Perform one-point normalization	yes
Normalization mass	176 (internal standard Fluorene-D10)
Normalization time D1 (sec)	5314.71
Normalization time D2 (sec)	4.14

Table S 9: *GC oven temperature profile for gas phase analysis by GC–MS*

T rate [°C min^{-1}]	T [°C]	Holding time [min]
-	60	6
5	300	5

Table S 10: *Mass spectrometric parameters for gas phase analysis by GC–MS*

MS Parameter	Setting
Scan rate	3.3 Hz
Mass acquisition range	35 – 500 Daltons (Da)
Electron ionization energy	70 eV
Ion source temperature	230 °C
Interface temperature	250 °C

Table S 11: *Compounds used for the internal standard (ISTD) and calibration standard mixture for gas phase analysis by GC–MS. The performed quantitation method has been previously published.*[2, 3]

ISTD (m/z)	Concentration [g L^{-1}]	Calibration Standard Mixture (m/z)	Precision [% RSD]
Benzene d6 (84)	0.2370	Benzene (78)	19
Toluene d8 (98)	0.2344	Toluene (91)	3
o-Xylene d10 (98)	0.0787	m-Xylene (91)	8
Naphthalene d8 (136)	0.0797	o-Xylene (91)	7
Biphenyl d10 (164)	0.0719	Ethylbenzene (91)	11
Acenaphthylene d8 (160)	0.0288	Styrene (104)	7
Acenaphthene d10 (164)	0.0149	Phenol (94)	17
Fluorene d10 (176)	0.0310	Indene (115)	12
Phenanthrene d10 (188)	0.0217	Indane (117)	15
Anthracene d10 (188)	0.0241	Naphthalene (128)	15
n-Heptane d16 (66)	0.0664	Fluorene (166)	14
n-Dodecane d26 (66)	0.0693	Acenaphthene (154)	16
n-Hexadecane d34 (66)	0.0387	Acenaphthylene (152)	12
		Phenanthrene (178)	9
		Anthracene (178)	7
		1-Methylnaphthalene (142)	17
		2-Methylnaphthalene (142)	14
		n-Alkanes, C8-C20 (57)	

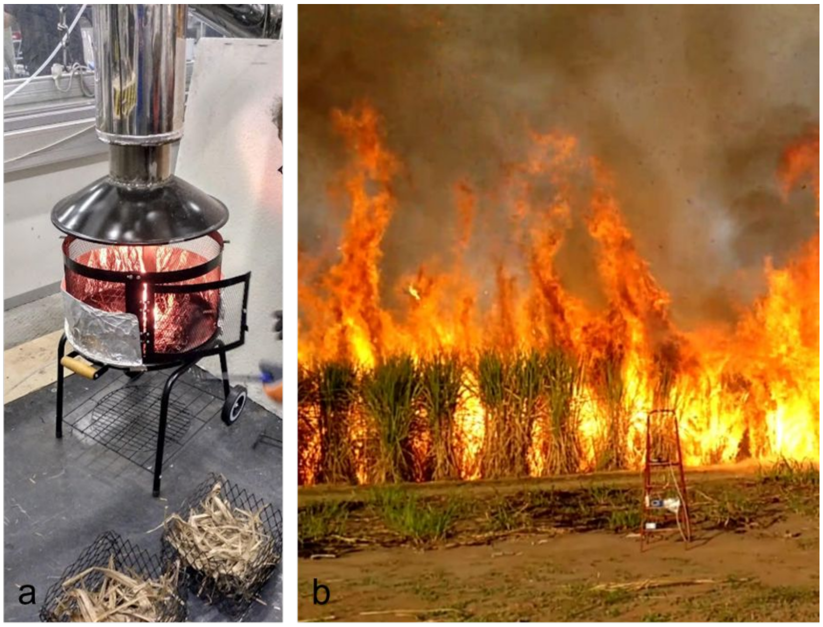

Figure S 2: *Photographs from **a)** the batch-wise burning of dried sugarcane leaves during the laboratory experiments and **b)** the open-field sugarcane burning experiments in South Africa*

Table S 12: *Key tracers for organic components in BBOA found in literature used for targeted evaluation.*

Peak no.	Compound name	Class	RT1 [sec]	RT2 [sec]	Literature
1	Hexadecane	Alkanes	5209.72	2.58	[4]
2	Heptadecane	Alkanes	5539.7	2.65	
3	Nonadecane	Alkanes	6144.66	2.72	
4	Eicosane	Alkanes	6419.64	2.77	
5	Heneicosane	Alkanes	6684.62	2.81	
6	Docosane	Alkanes	6939.61	2.76	
7	Tricosane	Alkanes	7154.59	2.61	
8	Tetracosane	Alkanes	7324.58	2.55	
9	Pentacosane	Alkanes	7469.57	2.52	
10	Hexacosane	Alkanes	7594.57	2.52	
11	Heptacosane	Alkanes	7704.56	2.53	
12	Octacosane	Alkanes	7804.55	2.55	
13	Nonacosane	Alkanes	7894.55	2.58	
14	Triacontan	Alkanes	7979.54	2.65	
15	Hentriacontan	Alkanes	8054.54	2.78	
16	Dotriacontan	Alkanes	8139.53	2.92	
17	Tritriacontan	Alkanes	8224.52	3.08	
18	Tetratriacontane	Alkanes	8314.52	3.26	
19	Pentatriacontane	Alkanes	8419.51	3.46	
20	Hexatriacontane	Alkanes	8529.51	3.71	
21	Furfural	Furan derivatives	1924.93	3.88	[5, 6]
22	2(5H)-Furanone, 3-methyl-	Furan derivatives	2739.88	4.34	[7]
23	Phenol, 2-methoxy-	Methoxyphenols	3264.84	3.70	[7, 8]
24	Creosol	Methoxyphenols	3734.81	3.61	[7, 8]

**25**	Phenol, 2,6-dimethoxy-	Methoxyphenols	4399.77	4.13	7, 8
**26**	Vanillin	Methoxyphenols	4619.76	4.34	4, 8, 9
**27**	Vanillic Acid	Methoxyphenols	4749.75	4.02	4, 8-10
**28**	Acetovanillone	Methoxyphenols	4934.74	4.31	8, 9
**29**	Guaiacylacetone	Methoxyphenols	5069.73	4.34	9
**30**	Syringaldehyde	Methoxyphenols	5524.7	4.74	4, 8, 9
**31**	Acetosyringone	Methoxyphenols	5744.68	4.71	4, 8, 9
**32**	Levoglucosenone	Monosaccharide derivatives	3414.83	4.35	5, 11, 12
**33**	1,4:3,6-Dianhydro-α-d-glucopyranose (Isomer 1)	Monosaccharide derivatives	3814.81	4.36	4
**34**	1,4:3,6-Dianhydro-α-d-glucopyranose (Isomer 2)	Monosaccharide derivatives	3879.8	4.36	4
**35**	Levoglucosan	Monosaccharide derivatives	4989.73	4.85	4, 9, 10
**36**	9H-Fluoren-9-one	O-PAH	5864.68	4.76	13, 14
**37**	1H-Phenalen-1-one	O-PAH	6389.64	0.59	
**38**	6H-Benz[de]anthracen-6-one	O-PAH	7644.56	4.25	
**39**	Naphthalene	PAH	3764.81	3.74	1, 13
**40**	Naphthalene, 1-methyl-	M-PAH	4234.78	3.69	1, 13
**41**	Naphthalene, 2-methyl-	M-PAH	4299.78	3.79	1, 13
**42**	Acenaphthylene	PAH	4859.74	4.20	1, 10, 13
**43**	Acenaphthene	PAH	5024.73	4.44	1, 10, 13
**44**	Phenanthrene	PAH	6004.67	4.76	1, 10, 13
**45**	Anthracene	PAH	6039.66	4.67	1, 10, 13
**46**	Fluoranthene	PAH	6819.61	0.15	1, 9, 10, 13
**47**	Pyrene	PAH	6974.6	0.04	1, 9, 10, 13
**48**	Benz[a]anthracene	PAH	7574.57	4.03	1, 9, 10, 13
**49**	Chrysene	PAH	7589.57	4.09	1, 9, 10, 13
**50**	Benzo[b]fluoranthene	PAH	7939.54	4.19	1, 9, 10, 13
**51**	Benzo[k]fluoranthene	PAH	7964.54	4.16	9, 10, 13
**52**	Benzo[e]pyrene	PAH	8014.54	4.61	9
**53**	Benzo[a]pyrene	PAH	8029.54	4.68	1, 9, 10, 13
**54**	Perylene	PAH	8049.54	4.75	9, 13
**55**	Dibenz[a,h]anthracene	PAH	8304.52	1.15	1, 9, 13
**56**	Indeno[1,2,3-cd]fluoranthene	PAH	8314.52	1.20	13
**57**	Indeno[1,2,3-cd]pyrene	PAH	8344.52	1.41	9, 10, 13
**58**	Benzo[ghi]perylene	PAH	8429.51	2.26	1, 10, 13
**59**	Campesterol	Phytosterols	8064.54	3.39	4
**60**	Stigmastan-3,5-diene	Phytosterols	8144.53	3.57	4
**61**	α-Amyrone	Triterpenoids	8424.51	0.02	4
**62**	β-Amyrone	Triterpenoids	8489.51	0.35	4
**63**	Biphenyl	Other	4559.76	3.80	13
**64**	2-Cyclopenten-1-one	Other	1979.92	3.92	5

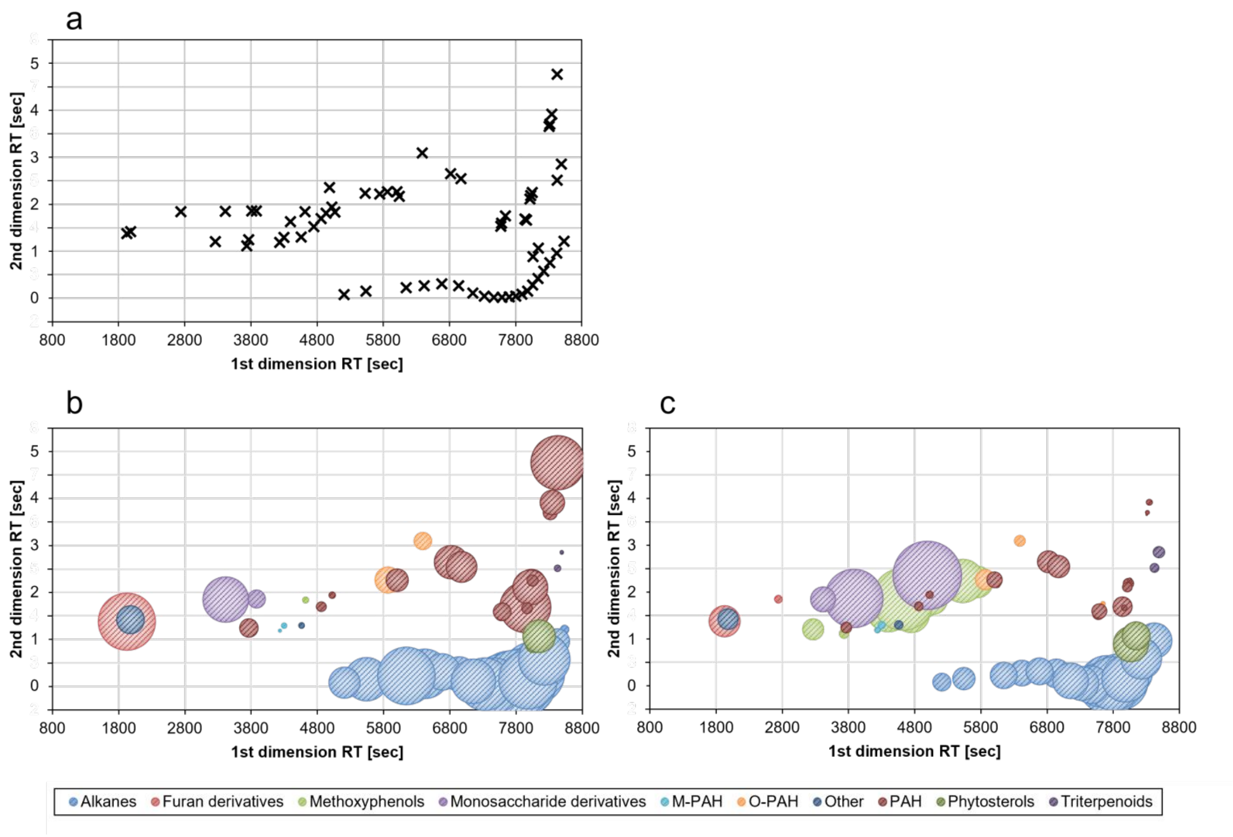

Figure S 3: a) *Targeted analysis of 64 marker compounds from biomass burning found in TD–GC×GC–TOFMS measurements of sugarcane burning emissions (compounds are shown in Table S1);* ***b)*** *Classification of referenced compounds in a GCxGC contour plot of sugarcane burning* *chamber* *experiments;* ***c)*** *Classification of referenced compounds in a GCxGC contour plot of sugarcane burning* *field* *experiments (TIC normalized areas depicted as size of bubbles)*

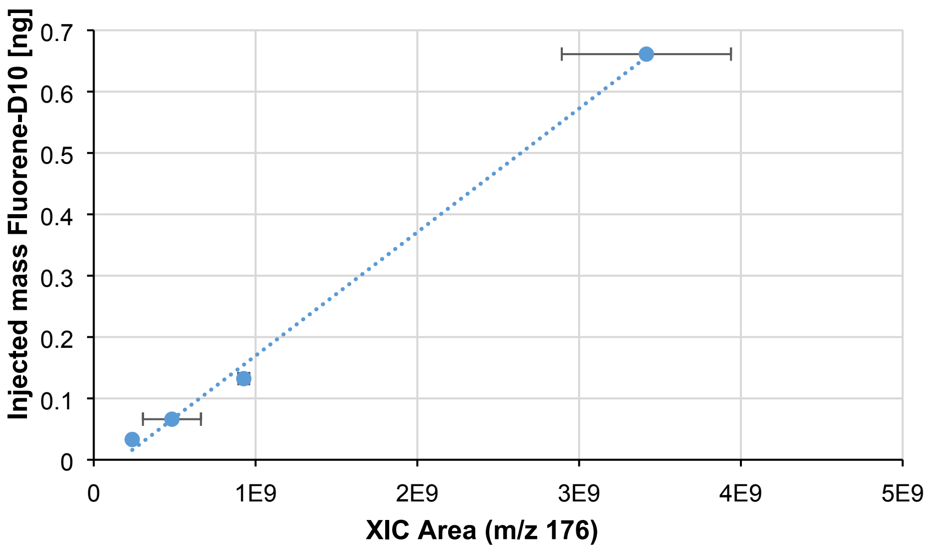

Figure S 4: *For the semi-quantification of the targeted reference particle phase compounds by TD-GC×GC-TOFMS (see* ***Table S12****), the concentrations (ng* m^{-3}*) were derived from a 4-point calibration using the internal standard compound fluorene-D10. The total ion chromatogram (TIC) area from each respective compound was used. Values were normalized to the corresponding sampling volume and were corrected for the respective dilution factor (field experiments = n.a. (set to 1) and laboratory experiments = 6). Furthermore, the respective effective filter diameter (42 mm) was considered from which a 10 mm punch was used during the measurements.*

Table S 13: *Compounds derived from the non-targeted analysis with positive fold changes and a significance level of p<0.01.*

**Peak no.**	**Compound name**	**Class**	**Subclass**	**Functional group**	**RT1 [sec]**	**RT2 [sec]**
**1**	Pyridine, 2-(2-methylpropyl)-	Other	Heterocyclic/-aromatic	Pyridines	3044.9	3.2
**2**	1,4-Benzenediol, 2,5-bis(1,1-dimethylpropyl)-	Methoxyphenols			6389.6	4.0
**3**	Pyridine, 2-ethyl-6-methyl-	Other	Heterocyclic/-aromatic	Pyridines	2909.9	3.4
**4**	Vanillin	Methoxyphenols			4619.8	4.4
**5**	1-Dodecanol, 3,7,11-trimethyl-	Other	Non-cyclic	Alcohols	5619.7	2.7
**6**	Quinoline	Other	Heterocyclic/-aromatic	Pyridines	4004.8	4.2
**7**	Phenol, 2,6-dimethoxy-	Methoxyphenols			4394.8	4.2
**8**	1,4:3,6-Dianhydro-α-d-glucopyranose	Monosaccharide derivatives			3879.8	4.4
**9**	7-Methyl-1-naphthol	O-PAH			4579.8	4.0
**10**	Cinnamaldehyde, β-methyl-	Other	Aromatic	Carbonyls	4184.8	4.0

**11**	9-Methyltricyclo [4.2.1.1(2,5)] deca-3,7-diene-9,10-diol	Other	Cyclic	Alcohols	4519.8	3.9
**12**	1H-Inden-1-one, 2,3-dihydro-	O-PAH			4179.8	4.2
**13**	4-(2,6,6-Trimethyl-cyclohex-1-enyl)-but-3-en-2-one oxime	Other	Cyclic	Oximes	5899.7	4.7
**14**	Heptadecane	n-alkanes			5539.7	2.7
**15**	1(2H)-Acenaphthylenone	O-PAH			5609.7	4.8
**16**	2(3H)-Benzofuranone, hexahydro-4,4,7a-trimethyl-	Furans			4554.8	3.8
**17**	Nonadecane	n-alkanes			6139.7	2.7
**18**	Heneicosane	n-alkanes			6684.6	2.8
**19**	2-Propenal, 3-phenyl-	Other	Aromatic	Carbonyls	3919.8	4.2
**20**	Tricyclo[3.3.1.0(2,8)]nona-3,6-dien-9-one	Other	Cyclic	Carbonyls	3939.8	4.1
**21**	Eicosane	n-alkanes			6419.6	2.8
**22**	2-Methoxy-4-vinylphenol	Methoxyphenols			4254.8	3.8
**23**	Benzene, 2-methoxy-4-(2-propenyl)-1-(1-propynyloxy)-	Other	Aromatic	Ethers	5314.7	4.1
**24**	Benzene, (1-ethyl-1-propenyl)-	Other	Aromatic	C_xH_y	3779.8	3.6
**25**	Fluoranthene	PAH			6819.6	0.2
**26**	Pyrene	PAH			6974.6	0.1
**27**	Pyridine, 2,3,6-trimethyl-	Other	Heterocyclic/-aromatic	Pyridines	3119.9	3.4
**28**	Octadecane	n-alkanes			5849.7	2.7
**29**	Docosane	n-alkanes			6939.6	2.8
**30**	D:C-Friedo-B':A'-neogammacer-9(11)-ene, 3-methoxy-, (3β)-	Triterpenoids			8509.5	0.2
**31**	D-(+)-Ribono-1,4-lactone, triacetate	Monosaccharide derivatives			4644.8	3.9
**32**	D:C-Friedo-B':A'-neogammacer-9(11)-ene, 3-methoxy-, (3β)-	Triterpenoids			8464.5	4.9
**33**	3-Eicosene	Other	Non-cyclic	C_xH_y	6674.6	2.9
**34**	1,2,4-Trimethoxybenzene	Other	Aromatic	Ethers	4749.7	4.1
**35**	1-Tricosene	Other	Non-cyclic	C_xH_y	7149.6	2.6
**36**	Benz[a]anthracene	PAH			7574.6	4.0
**37**	2-Isobutyl-4-methylpyridine	Other	Heterocyclic/-aromatic	Pyridines	3524.8	3.2
**38**	Benzofuran, 2,3-dihydro-	Furans			3854.8	3.7
**39**	Tricosane	n-alkanes			7154.6	2.6
**40**	Benzaldehyde, 4-hydroxy-3,5-dimethoxy-	Methoxyphenols			5524.7	4.8

**41**	Furan, 2-butyltetrahydro-	Furans			7934.5	2.8
**42**	1,4-Naphthalenedione, 2,3,6-trimethyl-	O-PAH			5894.7	4.3
**43**	D-Friedoolean-14-ene, 3-methoxy-, (3β)-	Triterpenoids			8304.5	4.3
**44**	2-Cyclopenten-1-one, 2,3-dimethyl-	Other	Cyclic	Carbonyls	3019.9	3.7
**45**	Phenol, 2-methoxy-5-(1-propenyl)-	Methoxyphenols			4779.7	3.8
**46**	5-tert-Butylpyrogallol	Methoxyphenols			5024.7	4.0
**47**	Phenol, 2-methoxy-	Methoxyphenols			3264.8	3.7
**48**	Ethanone, 1-(4-hydroxy-3,5-dimethoxyphenyl)-	Methoxyphenols			5744.7	4.8
**49**	Hexadecane, 2,6,10,14-tetramethyl-	Other	Non-cyclic	C_xH_y	5859.7	2.6

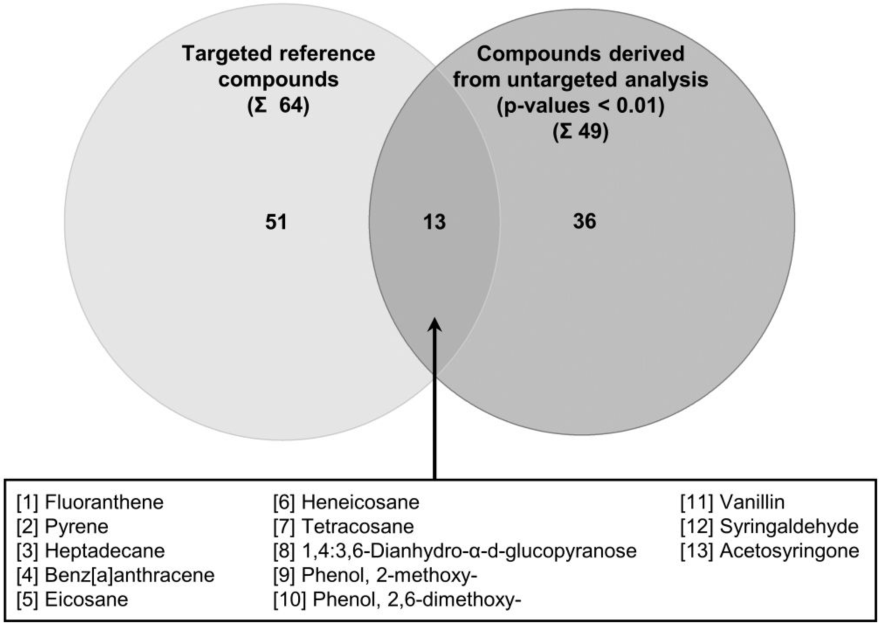

Figure S 5*: Venn diagram illustrating the intersection of chemical compounds obtained from two evaluation approaches: Targeted evaluation of 64 reference compounds (Table S12) and non-targeted evaluation using ChromaTOF Tile (Table S13).*

References

1. Geldenhuys, G.; Orasche, J.; Jakobi, G.; Zimmermann, R.; Forbes, P. B. C., Characterization of Gaseous and Particulate Phase Polycyclic Aromatic Hydrocarbons Emitted During Preharvest Burning of Sugar Cane in Different Regions of Kwa-Zulu Natal, South Africa. *Environmental Toxicology and Chemistry* **2023,** *42*, (4), 778-792.
2. Gawlitta, N.; Orasche, J.; Geldenhuys, G.-L.; Jakobi, G.; Wattrus, M.; Jennerwein, M.; Michalke, B.; Gröger, T.; Forbes, P.; Zimmermann, R., A study on the chemical profile and the derived health effects of heavy-duty machinery aerosol with a focus on the impact of alternative fuels. *Air Quality, Atmosphere & Health* **2023,** *16*, (3), 535-551.
3. Mason, Y. C.; Schoonraad, G.-L.; Orasche, J.; Bisig, C.; Jakobi, G.; Zimmermann, R.; Forbes, P. B. C., Comparative sampling of gas phase volatile and semi-volatile organic fuel emissions from a combustion aerosol standard system. *Environmental Technology & Innovation* **2020,** *19*, 100945.
4. Simoneit, B. R. T., Biomass burning — a review of organic tracers for smoke from incomplete combustion. *Applied Geochemistry* **2002,** *17*, (3), 129-162.
5. Moraes, M. S. A.; Georges, F.; Almeida, S. R.; Damasceno, F. C.; Maciel, G. P. d. S.; Zini, C. A.; Jacques, R. A.; Caramão, E. B., Analysis of products from pyrolysis of Brazilian sugar cane straw. *Fuel Processing Technology* **2012,** *101*, 35-43.
6. Andreae, M. O., Emission of trace gases and aerosols from biomass burning – an updated assessment. *Atmos. Chem. Phys.* **2019,** *19*, (13), 8523-8546.
7. Kumar, M.; Upadhyay, S. N.; Mishra, P. K., Pyrolysis of Sugarcane (Saccharum officinarum L.) Leaves and Characterization of Products. *ACS Omega* **2022,** *7*, (32), 28052-28064.
8. Liu, C.; Chen, D.; Chen, X. e., Atmospheric Reactivity of Methoxyphenols: A Review. *Environmental Science & Technology* **2022,** *56*, (5), 2897-2916.
9. Li, W.; Ge, P.; Chen, M.; Tang, J.; Cao, M.; Cui, Y.; Hu, K.; Nie, D., Tracers from Biomass Burning Emissions and Identification of Biomass Burning. *Atmosphere* **2021,** *12*, (11), 1401.
10. dos Santos, C. Y. M.; Azevedo, D. d. A.; de Aquino Neto, F. R., Selected organic compounds from biomass burning found in the atmospheric particulate matter over sugarcane plantation areas. *Atmospheric Environment* **2002,** *36*, (18), 3009-3019.
11. Lin, Y.-C.; Cho, J.; Tompsett, G. A.; Westmoreland, P. R.; Huber, G. W., Kinetics and Mechanism of Cellulose Pyrolysis. *The Journal of Physical Chemistry C* **2009,** *113*, (46), 20097-20107.
12. Worton, D. R.; Goldstein, A. H.; Farmer, D. K.; Docherty, K. S.; Jimenez, J. L.; Gilman, J. B.; Kuster, W. C.; de Gouw, J.; Williams, B. J.; Kreisberg, N. M.; Hering, S. V.; Bench, G.; McKay, M.; Kristensen, K.; Glasius, M.; Surratt, J. D.; Seinfeld, J. H., Origins and composition of fine atmospheric carbonaceous aerosol in the Sierra Nevada Mountains, California. *Atmos. Chem. Phys.* **2011,** *11*, (19), 10219-10241.
13. Samburova, V.; Connolly, J.; Gyawali, M.; Yatavelli, R. L. N.; Watts, A. C.; Chakrabarty, R. K.; Zielinska, B.; Moosmüller, H.; Khlystov, A., Polycyclic aromatic hydrocarbons in biomass-burning emissions and their contribution to light absorption and aerosol toxicity. *Science of The Total Environment* **2016,** *568*, 391-401.
14. Shen, G.; Xue, M.; Wei, S.; Chen, Y.; Wang, B.; Wang, R.; Lv, Y.; Shen, H.; Li, W.; Zhang, Y.; Huang, Y.; Chen, H.; Wei, W.; Zhao, Q.; Li, B.; Wu, H.; Tao, S., Emissions of parent, nitrated, and oxygenated polycyclic aromatic hydrocarbons from indoor corn straw burning in normal and controlled combustion conditions. *Journal of Environmental Sciences* **2013,** *25*, (10), 2072-2080.
